# Chewing Gum and Health: A Mapping Review and an Interactive Evidence Gap Map

**DOI:** 10.3390/nu17172749

**Published:** 2025-08-25

**Authors:** Aesha Allam, Silvia Cirio, Claudia Salerno, Nicole Camoni, Guglielmo Campus, Maria Grazia Cagetti

**Affiliations:** 1 Department of Biomedical, Surgical and Dental Sciences, University of Milan, 20142 Milan, Italy; silvia.cirio@unimi.it (S.C.); claudia.salerno@students.unibe.ch (C.S.); nicole.camoni@unimi.it (N.C.); maria.cagetti@unimi.it (M.G.C.); 2 Department of Restorative, Preventive and Pediatric Dentistry, School of Dental Medicine, University of Bern, 3012 Bern, Switzerland; 3 Dental Unit, Asst Valle Olona, 21052 Gallarate, Italy; 4 Department of Cariology, Institute of Odontology, Sahlgrenska Academy, University of Göteborg, 41390 Gothenburg, Sweden; guglielmo.giuseppe.campus@gu.se; 5 Department of Oral and Maxillofacial Sciences, Sapienza University of Rome, 00161 Rome, Italy; 6 Department of Cariology, Saveetha, Dental College and Hospitals, SIMATS, Chennai 600077, India

**Keywords:** chewing gum, well-being, surgical aid, medical aid, sports performance, evidence gap map, mapping review

## Abstract

**Background:** Chewing gum is a simple, accessible tool with high user compliance, traditionally associated with oral health benefits. Although its potential effects on different aspects of health and well-being, beyond its oral applications, have been explored, the area remains relatively under-researched. This mapping review and evidence gap map (EGM) aimed to evaluate the existing literature on the non-oral health applications of chewing gum and to identify gaps in the literature. **Methods:** A comprehensive search was conducted across five databases (Scopus, Embase, PubMed, PsycINFO, and CINAHL) using tailored search strategies. Abstracts were screened against predefined eligibility criteria using EPPI-Reviewer version 6, with full texts reviewed only when relevant information could not be drawn. The included studies were coded by gum type, outcome, and study design, and the EGM was constructed using EPPI-Mapper version 2.4.5. **Results:** Of the 2614 identified records, 1326 were screened after duplicate removal, and 260 studies were included in the final analysis. Three main areas of application emerged: for enhancing well-being and performance, as a medical aid and as a surgical/procedural aid. The EGM indicated that the most frequently studied uses of chewing gum were in sports performance, smoking cessation, and post-operative recovery. However, notable research gaps were found, particularly in paediatric and geriatric contexts. **Conclusions:** Chewing gum has been extensively studied as a surgical or procedural aid, particularly for post-operative gastrointestinal recovery, but its broader applications for well-being, performance, and its use in paediatric and elderly populations remain underexplored. Further high-quality research using standardised methodologies is needed to address these gaps.

## 1. Introduction

The use of chewing gum (CG) can be traced back to antiquity. The Egyptians and the Mayans, for instance, are known to have chewed natural resins, which are precursors of the CG that we recognise today [1]. Its modern form emerged in the 19th century, coinciding with the commercialisation of chicle-based gum, ultimately evolving into a global product of considerable cultural and economic impact [2]. CG is generally classified as a confectionery item and is composed primarily of gum base, which is an insoluble, non-nutritive colloidal system that is inert and indigestible, meaning that it does not dissolve during mastication [3]. Conventionally, the purpose of CG has been to provide a pleasant sensory experience through the addition of flavours and sweeteners. However, it has also been proposed that CG can be used as a therapeutic agent, both with and without the incorporation of active ingredients [4,5,6,7].

Historically, the use of CG has primarily been associated with oral health, particularly in caries prevention using non-fermentable sweeteners such as xylitol [8]. It has also been employed to alleviate conditions such as halitosis and xerostomia [9]. However, in recent decades, scientific interest in CG has expanded significantly beyond the dental field, encompassing a wide range of health and well-being domains [10,11,12].

Health is understood as the overall state of physical, mental, and social well-being, not merely the absence of disease or infirmity, as defined by the World Health Organization [13]. Well-being is considered to be a broader, multidimensional construct that reflects how people feel, how they function, and how they evaluate their lives in both personal and social domains [14].

Recent studies have investigated the role of CG in various health-related applications, including smoking cessation, enhancement of athletic performance, and post-operative recovery of gastrointestinal function [5,15,16]. The latter is believed to occur through mechanisms such as sham feeding, which activates the cephalic–vagal axis [17]. A growing body of research has examined the potential of CG to stimulate the neuromuscular system, regulate anxiety, enhance alertness, and support appetite control. These findings suggest that CG may have broader applications as a tool for promoting physical and mental well-being [11,18].

One of the key advantages of CG in the context of health promotion is its non-pharmacological nature. It is low-cost, easily accessible, and generally well accepted by patients, especially the younger ones, as it is not perceived as a conventional medical treatment. This is primarily attributed to its non-conventional status, which is perceived as less imposing and more acceptable by patients. It is evident that these features render this tool a potentially valuable asset in public health strategies, particularly with respect to preventive interventions and low-threshold therapeutic approaches [19].

Despite this mounting interest, a comprehensive and structured overview of the scientific literature regarding the non-oral health uses of CG remains elusive. To address this gap, a mapping review (MR) was conducted. An MR is a methodological approach designed to explore and describe a broad body of literature using predefined variables [20]. A fundamental distinction exists between systematic reviews, which are oriented towards intervention efficacy, and MRs, which are concerned with visually synthesising existing intervention areas. This objective is pursued by means of evidence gap maps (EGMs), which are presented in the form of bubble maps, multi-axis charts, and time-based visualisations. The purpose of this approach is to identify gaps in the research landscape.

In this context, the present MR, supported by an EGM, explores the use of CG in health and well-being by synthesising available studies and offering a concise overview to guide future research and public health efforts. Since the oral benefits of CG have already been extensively studied in previous reviews [21,22,23,24], they were excluded from the research question in order to focus on the effects of CG on any other aspects of health and well-being, domains for which comprehensive reviews are currently lacking in the literature.

## 2. Materials and Methods

### 2.1. PRISMA Statement and Protocol Registration

This review adhered to the Preferred Reporting Items for Systematic Reviews and Meta-Analyses (PRISMA) extension for scoping reviews [25], which can also be applied to MRs [26]. The complete checklist can be found in Supplementary Table S1.

The study protocol was registered at the OSF Registry (Open Science Framework); registration DOI: https://doi.org/10.17605/OSF.IO/JWDRX (accessed on 7 July 2025).

### 2.2. PICO Elements

The research question of the present review was as follows: “What are the research areas and type of evidence in which CG has been explored for its potential benefits to human health and well-being, apart from oral health?”

The research question followed the elements of the PICO model, which can be used for a MR [26], and included the following:

**P** (Population): Individuals of any age and sex, regardless of whether they are healthy or have a diagnosed pathological condition.
**I** (Intervention): Any type of CG, whether as a standalone intervention or in combination with other treatments.
**C** (Comparator): Any other types of interventions; studies evaluating CG without a comparator were included as well.
**O** (Outcome): Benefits on overall health and well-being, including those assessing effects in pathological conditions.

### 2.3. Search Strategy

Five databases were searched (Scopus, Embase, PubMed, PsycINFO, and CINAHL) up to 20 July 2025. For each database, customised search strings were constructed by adapting the Boolean operators and syntax to match the technical requirements of each platform (Supplementary Table S2). Filters regarding language (English) and date of publication (last 10 years) were applied.

### 2.4. Eligibility Criteria and Screening

The results of each database were then uploaded to the EPPI-Reviewer Web version 6 (EPPI-R) for screening [27]. Two reviewers (A.A. and S.C.) were trained and, before screening began, a pilot test was conducted to verify proper adherence to the eligibility criteria. After removing duplicates, records were assessed based on title and abstract by the two authors. Inter-author reliability was assessed as the percentage of agreement using Cohen’s kappa statistics.

Following the removal of duplicates, the articles were subjected to screening on the basis of title and abstract only; full texts were consulted solely in instances where the nature of the CG, the study outcome, or the study design was unclear.

The eligibility criteria were the following:

1. Any study investigating the use of any type of CG for health-related and well-being purposes, excluding oral health;
2. All types of studies except for case reports, case series, in vitro studies, study protocols, guidelines, letters to the editor, and conference abstracts;
3. Studies in which CG was used either alone or in combination with other interventions;
4. Studies regardless of the demonstrated efficacy of CG on the assessed outcomes.

### 2.5. Coding and EGM Development

For the coding process, each study was screened jointly by two authors (A.A. and S.C.), and disagreements were resolved through discussion; when this was not possible, a third author (M.G.C.) was consulted. The development of primary codes and subcodes for the outcome followed an iterative approach: they were not predefined but were progressively added and, when necessary, modified during the evaluation of studies to better adapt and accurately represent the characteristics of the different studies. In instances where an article was evaluated against multiple outcomes, it was common practice to assign more than one primary code and/or subcode. The same was applied for codes’ assignment for study design and CG type.

Using EPPI-Reviewer Web version 6 (EPPI-R) and its built-in coding tools, a coding framework (*code tree*) was developed to categorise the included studies. After inter-author discussion, three main (*code sets*) categories were identified: type of CG, outcome, and study design. For each category, primary codes (*parent codes*) were developed, and subcodes (*child codes*) were created when further specification was needed. After coding all included studies, a JSON file was exported and uploaded into EPPI-Mapper version 2.4.5 to generate the EGM [28].

## 3. Results

### 3.1. Database Search Results

**Figure 1:**
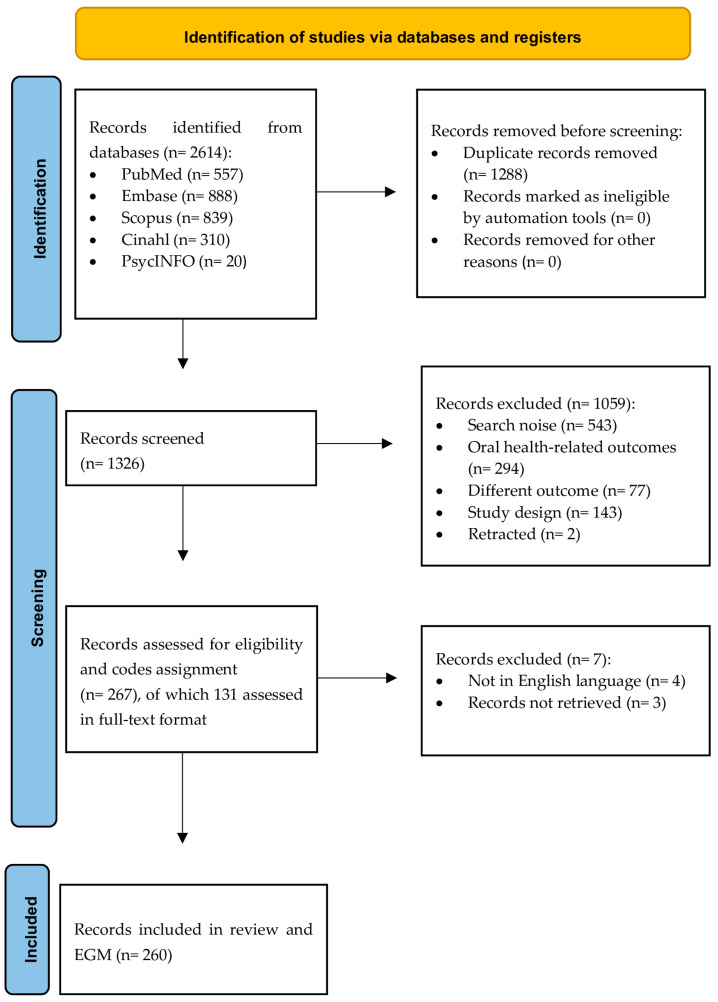
PRISMA flowchart of the selection process of the records.

The electronic search retrieved 2614 records, of which 1326 remained after removing duplicates. Following title and abstract screening, 1057 records were excluded for not addressing the research question, focusing on oral health outcomes, unrelated outcomes, or ineligible study designs. Two retracted articles were also excluded. Full texts of 131 records were assessed, with seven further exclusions due to unavailability or language. Ultimately, 260 records were included in the review and EGM (Figure 1; Supplementary Table S3). Cohen’s kappa for the interviewers’ agreement was 0.75.

### 3.2. Coding and EGM Development

Table 1 summarises the primary codes along with the number of studies categorised by study design and CG type, as well as the primary codes, subcodes, and the corresponding number of studies for each assessed outcome.

The primary codes related to the assessed outcomes were defined as follows:

- The Use of CG for Well-Being and Performance: Assigned to studies that investigated the use of CG as an intervention to enhance various aspects of human performance, including cognitive, physical, or other functional domains, in healthy subjects;
- The Use of CG as a Medical Aid: Assigned to studies that evaluated CG as a therapeutic or supportive intervention for medical conditions or symptoms;
- The Use of CG as a Surgical/Procedural Aid: Assigned to studies that examined CG as an adjunctive intervention intended to facilitate surgical or diagnostic procedures, enhance patient-centred outcomes, or accelerate post-operative recovery.

The primary codes for study design included the following:

- Randomised controlled trial (RCT);
- Non-RCT;
- Surveys;
- Reviews and Meta-Analyses.

The primary codes for CG types were assigned as follows:

- Sugar-Free: CGs declared as sugar-free or containing polyols other than xylitol.
- Sugar-Free with Xylitol: Sugar-free CGs specifically containing xylitol.
- Sugared: CGs containing sugar.
- With Caffeine: CGs containing caffeine.
- With Nicotine: CGs containing nicotine.
- With Other Bioactive Ingredients: CGs containing bioactive compounds other than nicotine or caffeine.
- Not Specified: CGs reported without details on their ingredients.

The decision to create a separate code for “sugar-free with xylitol” rather than including it in the broader “sugar-free” category was based on the considerable number of studies (n = 31) specifically investigating xylitol-containing CGs. A similar approach was applied to “with caffeine” and “with nicotine,” which were distinguished from the broader “with other bioactive ingredients” category due to the substantial volume of research in these specific areas.

The included studies with the assigned codes were mapped onto an EGM available online through the following link: https://aeshaallam.github.io/chewinggumandhealthmappingreviewandEGM/ accessed on 25 August 2025).

### 3.3. Studies’ Characteristics

**Figure 2:**
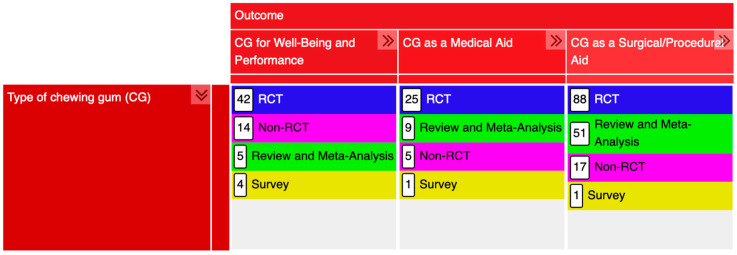
Screenshot of the compressed EGM, illustrating the distribution of the included records according to the study design.

Of the 260 included studies, 155 were RCTs, 36 non-RCTs, 65 reviews or meta-analyses, and 6 surveys, as shown in Figure 2. One study contained both a short report and an updated review and was assigned to both the non-RCTs and reviews/meta-analyses codes [29]; another study included both an RCT study and a literature review and, thus, was assigned to both primary codes of the study design [30].

**Figure 3:**
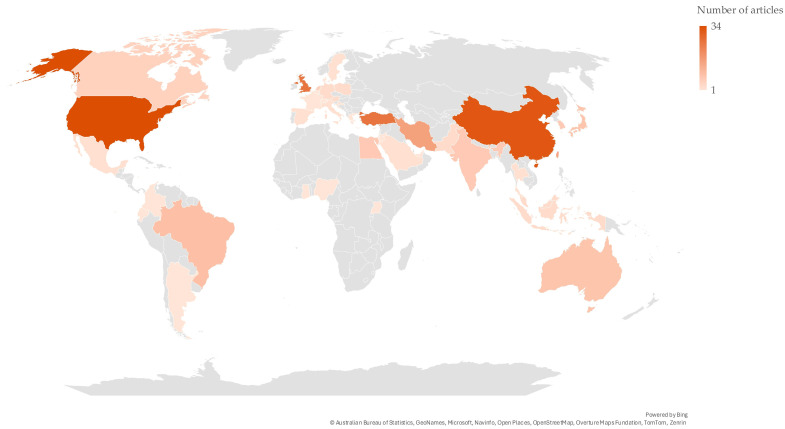
Map showing the geographical distribution of the included records by country.

Figure 3 illustrates the distribution of the included records by country. Most of the records originated from the United States (34), followed by China (32), the United Kingdom (26), and Turkey (26).

**Figure 4:**
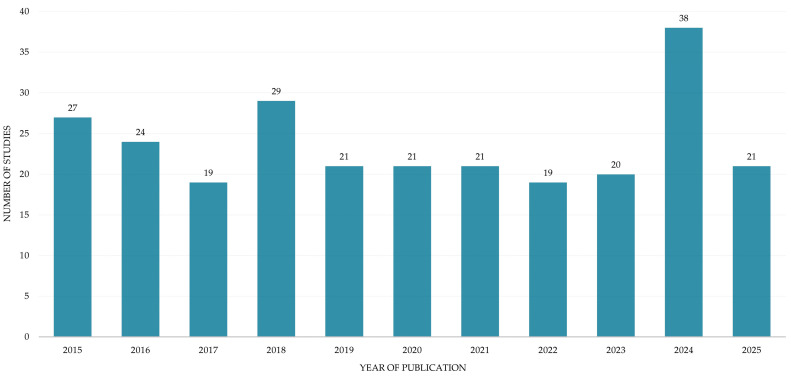
Annual distribution of the included studies. Data for 2025 are partial and reflect publications up to July 20th.

With regard to the year of publication, the included studies were published from 2015 to 2025. The year 2024 accounted for the highest number of studies (n = 38), whereas in the preceding years the number of studies was not only lower but also relatively stable (Figure 4).

### 3.4. Different Uses of CG

#### 3.4.1. CG for Well-Being and Performance

Among the included records, 65 investigated the use of CG for well-being and performance. Of these, 28 focused on its potential to enhance sports performance, 19 examined its effects on mental performance, 2 explored its impact on auditory processing, 14 assessed its role in anxiety, stress, and mood modulation, and 9 addressed metabolic regulation. Additionally, one study each evaluated its application in motion sickness and eyestrain. Interestingly, most studies on sports performance (n = 27) focused on the use of CG with caffeine, suggesting that caffeine may substantially contribute to performance enhancement in athletic contexts. Caffeinated CG has been shown to reduce fatigue [31,32,33], allowing athletes to sustain high levels of physical exertion for longer periods. Additionally, it also contributes to improvements in endurance, strength, and reaction time, supporting its role as a valuable ergogenic aid across multiple sports disciplines [12,32,33,34,35]. Only five reviews were published in this category (Figure 2), four of which focused on the use of CG with caffeine to enhance sports performance [5,36,37,38], while the remaining review focused on the use of CG for stress management and its effects on cognition [11].

#### 3.4.2. CG as a Medical Aid

A total of 40 studies investigated the use of CG as a medical aid. The largest subset (n = 14) focused on its role as a therapeutic aid for smoking cessation when containing nicotine. A plethora of studies have demonstrated the efficacy of nicotine gum as a standalone nicotine replacement therapy [4,39]. The potential of combining nicotine gum with other nicotine replacement therapies, such as nicotine patches and electronic cigarettes, has also been investigated for its role in enhancing adherence to smoking cessation [40,41,42]. Other applications in this category included thirst relief (n = 8) in patients undergoing haemodialysis or living with chronic heart failure, and comfort enhancement during pregnancy and childbirth (n = 6). Additionally, two studies explored its use for pain relief, and four examined its role in the management of gastrointestinal disorders. Two studies focused on the reduction in symptoms related to Ear, Nose, and Throat (ENT) conditions, including a review on the use of xylitol for managing otitis media in children. Additionally, one study investigated the effects of CG on glycaemic control in women with gestational diabetes, another examined its impact on cognitive function in individuals with Alzheimer’s disease, and one study explored its influence on attention in individuals with Attention Deficit Hyperactivity Disorder (ADHD). One study focused on the effects of gum on anthropometric indices and blood pressure.

#### 3.4.3. CG as a Surgical/Procedural Aid

A total of 155 studies evaluated the use of CG as an adjunct in surgical or procedural settings. The included studies analysed a range of outcomes related to both the pre-operative and post-operative phases.

A total of nine studies were conducted in the pre-operative period, with a focus on the effects of CG administration prior to endoscopic procedures. These studies highlighted two notable findings: firstly, they demonstrated an improvement in gastric visibility, and secondly, they indicated a reduced need for flushing. Furthermore, the studies indicated a low risk of aspiration, thereby confirming the safety of CG administration before such procedures. Six studies focused on the role of CG during pre-operative fasting. Additionally, five studies explored its use as a strategy to manage pre-operative anxiety, yielding inconsistent results.

In the post-operative period, the most frequently assessed outcome was gastrointestinal recovery, a focus that was evident in 116 studies. This outcome was often examined in relation to the length of hospital stay, reported in 26 studies. Collectively, the findings indicated that the administration of CG following surgery had a positive impact on gastrointestinal function, contributing to faster recovery and a reduction in both post-operative gastrointestinal complications (such as ileus) and hospital stay. A number of additional post-operative outcomes were also investigated, including post-operative nausea and vomiting, assessed in 15 studies; pain relief during and after procedures, reported in 13 studies; and patient satisfaction, evaluated in six studies. A smaller number of studies focused on more specific outcomes, such as post-operative sore throat (n = 5), appetite loss (n = 2), tongue pressure following esophagectomy (n = 1), and post-operative thirst (n = 1).

This category was found to contain the highest number of reviews and meta-analyses among all categories, with a total of 51 studies.

### 3.5. Study Population

Most of the records focused on the adult population (n = 244). Twelve studies investigated the use of CG in children, the majority of which (n = 11) addressed its role as a surgical/procedural aid [6,7,10,30,43,44,45,46,47,48,49], as shown in Table 2. Only one study on the use of CG as a medical aid investigated the use of CG with xylitol for acute otitis media [50]. In contrast, no studies were identified investigating the potential application of CG for well-being or performance enhancement.

Regarding the elderly population, only four studies were identified. In the well-being and performance category, one study examined the effects of CG on recognition memory in older adults [18]. Within the medical aid category, one study investigated the efficacy of olibanum gum in improving cognitive function in patients with mild-to-moderate Alzheimer’s disease [51]. The remaining two studies, categorised as CG for surgical or procedural aids, assessed the impact of CG on post-operative gastrointestinal recovery [15,52]. A summary of these studies is provided in Table 3.

## 4. Discussion

This MR offers a comprehensive overview of the scientific literature investigating the use of CG in health and well-being domains beyond oral health. The findings highlight an escalating interest in the therapeutic, supportive, and performance-enhancing roles of CG across diverse populations and clinical contexts.

In the development of the EGM, assigning primary codes required careful judgment due to frequent conceptual overlaps. For instance, smoking cessation interventions were challenging to categorise, as smoking reflects both a lifestyle factor and an addiction, the latter being clinically recognised as a disease [53]. Accordingly, these studies were assigned to the “CG as a medical aid” code. Similarly, anxiety was categorised based on context: pre-operative anxiety was coded under “CG as surgical/procedural aid,” whereas anxiety related to academic performance was classified as “CG for well-being and performance.” Studies on cognitive function in older adults were also classified by diagnostic status. Those involving individuals with dementia or Alzheimer’s disease were assigned to “CG as a medical aid,” while studies on cognitively healthy elderly participants were placed under “CG for well-being and performance”.

The remarkable versatility of CG is evident from its application across a wide range of applications. The highest number of studies was found in the context of surgical and procedural support, particularly for enhancing post-operative gastrointestinal recovery. The results reported in this area, including reduced ileus and decreased hospitalisation times, indicate that CG may be a straightforward, cost-effective intervention to facilitate recovery, particularly in abdominal surgeries [6,29,48]. CG can stimulate the passage of flatus and stool by activating the cephalic–vagal reflex, which improves gut motility. It is considered to be a form of sham feeding because the act of chewing mimics actual food intake without ingestion. Sham feeding has been shown to increase serum concentrations of the hormone gastrin, as well as duodenal alkaline and pancreatic secretions, thereby supporting the physiological basis for CG’s role in accelerating gastrointestinal recovery [54,55].

The medical aid category also revealed significant applications, particularly in smoking cessation. In this field, nicotine-containing gums were found to have a considerate amount of studies supporting their use [4,39,40,41]. Further studies have explored the integration of this strategy with other nicotine replacement strategies, with a view to improving adherence and success rates. Research on cessation medication has demonstrated that adherence to prescribed dosing and duration of medication is only partial, with approximately half or fewer smokers complying with the recommended guidelines. It is acknowledged that adherence typically diminishes over time; therefore, enhancing adherence may facilitate the promotion of long-term abstinence [56,57,58].

Other promising yet less-explored applications included the alleviation of symptoms in chronic conditions, the management of symptoms in ENT disorders [50,59], and cognitive support in conditions such as Alzheimer’s disease and ADHD [51,60], implying potential for further clinical investigation.

In the well-being and performance category, research has predominantly concentrated on the utilisation of caffeinated creatine monohydrate to enhance athletic performance, thereby substantiating its ergogenic capabilities [12,32,37]. Caffeine primarily works by blocking adenosine receptors, reducing fatigue and increasing alertness and muscle contractility [61]. These findings align with broader research on caffeine’s performance-enhancing properties and point to CG as a user-friendly alternative delivery method [62]. Fewer but noteworthy studies explored mental performance, anxiety reduction, and mood regulation, areas that may benefit from more rigorous and standardised investigations.

The predominance of RCTs within the included studies suggests a robust methodological foundation in numerous research domains. However, a considerable proportion of the extant literature also consists of narrative reviews, non-RCTs, and observational studies, particularly in contexts where CG is used as a surgical or procedural aid. To further strengthen future research quality and reporting, the adoption of standardised frameworks such as CONSORT for clinical trials and TIDieR for detailed intervention descriptions would be beneficial.

Despite the comprehensive nature of the MR, there are specific domains that have received insufficient exploration. For instance, although preliminary evidence suggested the potential of CG to enhance well-being and performance, this area was represented by only a small subset of studies. Furthermore, the utilisation of applications in paediatric and geriatric populations was found to be constrained, with most studies concentrating on adult subjects. Given the safety and high acceptability of CG, particularly among children, targeted research in these subgroups could offer promising opportunities for preventive and therapeutic interventions [63].

A salient finding is the potential of CG as a comprehensive and adaptable health instrument. The intervention’s affordability, accessibility, and non-pharmacological approach render it especially well suited for initiatives focused on enhancing health determinants and fostering disease control at both the individual and community levels. Moreover, its high acceptability and user-friendliness render it an ideal vehicle for delivering health-promoting agents or supporting behavioural changes that enhance overall well-being.

However, in just under one-third of the included studies (for detail, see Table 1 and https://aeshaallam.github.io/chewinggumandhealthmappingreviewandEGM/ accessed on 25 August 2025), the specific type or formulation of CG used was not clearly described, limiting reproducibility and comparability. Additionally, the potential existence of active ingredients, such as xylitol, may have elicited supplementary effects that extend beyond the scope of the present study. Future research should clearly report the composition of the CG used and ensure consistency across studies to facilitate meaningful comparisons and support meta-analytical synthesis.

To the best of the authors’ knowledge, this is the first MR providing readers with a comprehensive overview of the different applications of CG in health, a topic that remains relatively unfamiliar to many clinicians. This paper includes a substantial number of studies and offers an interactive EGM to enhance accessibility and facilitate the consultation of individual studies.

A significant strength of this review is its comprehensive scope, encompassing multiple health domains, along with its utilisation of an EGM to visually synthesise the findings. This format facilitates the identification of areas that are supported by robust evidence, while simultaneously drawing attention to topics that require further exploration.

However, it is imperative to acknowledge several limitations inherent to this approach. Firstly, the exclusion of non-English full-text articles may have resulted in the omission of relevant research. Furthermore, the 10-year time limit may have excluded certain areas of application: for example, the use of xylitol-containing CG in otorhinolaryngological conditions, where only systematic reviews were captured, but not individual clinical trials. Additionally, the exclusion of qualitative studies may have limited insights into user experiences, behavioural aspects, and contextual factors related to CG use. Finally, although the MR is valuable for illustrating the breadth of available evidence, it does not assess the quality or strength of that evidence; thus, a complementary systematic review would be necessary to evaluate these aspects in depth.

Research should be expanded by performing studies at the community level, especially including high-risk or underrepresented populations, such as children and the elderly, who may particularly benefit from a simple, cheap, and well-tolerated intervention like CG.

Promising preliminary results in the emerging area of well-being and performance highlight the need for further investigation through more consistent and evidence-based study designs. Furthermore, studies that explore the biological and psychological pathways through which CG employs its effects could facilitate the development of more targeted applications.

## 5. Conclusions

This MR underscores the substantial scope and growing scientific interest in the use of CG across diverse health and well-being domains, extending well beyond its traditional role in oral health. The most frequently studied application was CG as a surgical/procedural aid, particularly for post-operative gastrointestinal recovery and reduction in hospital stay. Less frequently investigated but noteworthy uses in this category included CG for managing pre-operative anxiety, preparation for endoscopy, post-operative pain relief, and support during pre-operative fasting.

The second most explored category was CG for well-being and performance, with a prominent focus on caffeinated CG for enhancing physical performance in sports. Other, less represented applications in this category included CG for cognitive enhancement and stress or anxiety management. The least examined category was CG as a medical aid, with nicotine-containing CG for smoking cessation being the most common application. Other uses, such as pain relief, thirst management, and treatment of gastrointestinal disorders, were comparatively underexplored.

Finally, CG use in paediatric and elderly populations remains markedly underrepresented across all categories, highlighting the need for further targeted research in these age groups.

## Figures and Tables

**Table 1:** Primary codes and subcodes assigned to the included records.

	Primary Code (n Studies)	Subcodes (n Studies)
**Assessed Outcomes**	CG for Well-Being and Performance (65)	Sports performance (28)
		Mental performance (19)
		Auditory processing (2)
		Anxiety, stress, and mood (14)
		Metabolic regulation (9)
		Motion sickness (1)
		Eyestrain (1)
	CG as a Medical Aid (40)	Smoking cessation (14)
		Thirst relief (8)
		Pregnancy and birth experience (6)
		Pain relief (2)
		GIT disorders (4)
		ENT disorders (2)
		Glycaemic control (1)
		Cognition in Alzheimer’s disease (1)
		Attention in ADHD (1)
		Cardiac disorders (1)
	CG as a Surgical/Procedural Aid (155)	Post-operative gastrointestinal recovery (116)
		Length of hospital stay (26)
		Post-operative nausea or vomiting (15)
		Pain relief intra- and post-operative/procedure (13)
		Pre-endoscopy (9)
		Pre-operative fasting (6)
		Patient satisfaction pre- and post-operative (6)
		Pre-operative anxiety (5)
		Post-operative sore throat (5)
		Post-operative appetite loss (2)
		Tongue pressure after esophagectomy (1)
		Post-operative thirst (1)
**Study Design**	RCT (155)	
	Non-RCT (36)	
	Survey (6)	
	Review and Meta-analysis (65)	
**Chewing Gum Type**	Sugar-free (97)	
	Sugar-free with xylitol (31)	
	Sugared (17)	
	With caffeine (34)	
	With nicotine (18)	
	With other bioactive ingredients (11) Not specified (78)	

CG: chewing gum; GIT: gastrointestinal tract; ENT: Ear, Nose, and Throat, ADHD: Attention Deficit Hyperactivity Disorder; RCT: Randomised Controlled Trial.

**Table 2:** Records that evaluated the use of CG in children.

First Author, Year	Study Design	CG Type	Outcomes (Primary Code and Subcode)
*CG as a medical aid*			
Azarpazhooh et al., 2016 [50]	Review/meta-analysis	Sugar-free with xylitol	ENT disorders
*CG as a surgical/procedural aid*			
Chan et al., 2017 [10]	RCT	Not specified	Post-operative gastrointestinal recovery
Fung et al., 2024 [6]	Review/meta-analysis	Not specified	Post-operative gastrointestinal recovery
Harrison et al., 2015 [43]	Review/meta-analysis	Sugared	Pain relief intra- and post-operative/procedure
Jennings et al., 2015 [44]	RCT	Sugar-free	Post-operative gastrointestinal recovery
Keefe et al., 2018 [45]	Review/meta-analysis	Not specified	Pain relief intra- and post-operative/procedure
			Post-operative nausea or vomiting
Koksoy et al., 2025 [46]	RCT	Sugar-free	Pain relief intra- and post-operative/procedure
López-Jaimez et al., 2016 [47]	RCT	Sugar-free	Post-operative gastrointestinal recovery
			Length of hospital stay
Meng et al., 2018 [30]	RCT	Sugar-free	Post-operative gastrointestinal recovery
Naz et al., 2023 [7]	RCT	Sugar-free	Post-operative nausea or vomiting
			Length of hospital stay
Tong et al., 2023 [48]	Review/meta-analysis	Not specified	Post-operative nausea or vomiting
Yildizeli et al., 2020 [49]	RCT	Not specified	Pain relief intra- and post-operative/procedure
			Pre-operative anxiety

CG: chewing gum; RCT: Randomised Controlled Trial; ENT: Ear, Nose, and Throat.

**Table 3:** Records that evaluated the use of CG in elderly populations.

First Author, Year	Study Design	CG Type	Outcomes (Primary Code and Subcode)
*CG for well-being and performance*			
Kim et al., 2019 [18]	Non-RCT	Sugar-free	Mental performance
*CG as a medical aid*			
Ghorat et al., 2024 [51]	RCT	With other bioactive ingredients (olibanum)	Cognition in Alzheimer’s disease
*CG as a surgical/procedural aid*			
Cha, Y.-H. et al., 2021 [15]	RCT	Sugar-free with xylitol	Post-operative gastrointestinal recovery
Du et al., 2021 [52]	Non-RCT	Sugar-free	Post-operative gastrointestinal recovery

CG: chewing gum; RCT: Randomised Controlled Trial.

## Data Availability

The data supporting the findings of this study are publicly available in the University of Milan Dataverse repository at the following DOI: https://doi.org/10.13130/RD_UNIMI/KP222G accessed on 7 July 2025. All datasets generated and analysed during the current study have been deposited and can be accessed.

## Supplementary Materials

The following supporting information can be downloaded at https://www.mdpi.com/article/10.3390/nu17172749/s1: Table S1: PRISMA-ScR checklist; Table S2: Search strings used for each searched database; Table S3: Included records.

## Abbreviations

The following abbreviations are used in this manuscript:

MR	Mapping Review
EGM	Evidence Gap Map
CG	Chewing Gum
OSF	Open Science Framework
RCT GIT	Randomised Controlled Trial Gastrointestinal Tract
ENT	Ear, Nose, and Throat
ADHD	Attention Deficit Hyperactivity Disorder
