# Supplementary material for: Chewing Gum and Health: A Mapping Review and an Interactive Evidence Gap Map
Source: Nutrients. 2025 Aug 25;17(17):2749. doi: 10.3390/nu17172749 (PMC12430410; doi:10.3390/nu17172749)
Supplement: Supplementary file 1 [file nutrients-17-02749-s001.zip › Table S2.pdf]

Table S2. Search strings used for each searched database.

PubMed	(((chewing gum[MeSH Terms]) OR ((((sugar free[Title/Abstract]) OR (sugar-free[Title/Abstract]) OR (sugarless[Title/Abstract])) AND (chewing gum*[Title])) OR (gum chewing[Title])))) AND ((((((((((((((((diseases category[MeSH Terms]) OR (surgery[Title/Abstract])) OR (surgical[Title/Abstract])) OR (post ADJ2 operative[Title/Abstract]))) OR (peri ADJ2 operative[Title/Abstract])) OR (pre ADJ2 operative[Title/Abstract])) OR (prevent*[Title/Abstract])) OR (therap*[Title/Abstract])) OR (health care category[MeSH Terms])) OR (functional stat*[Title/Abstract])) OR (pregnan*[Title/Abstract])) OR (delivery[Title/Abstract])) OR (mental performance[Title/Abstract])) OR (health outcome*[Title/Abstract])) OR (well being[Title/Abstract]))
Embase	'chewing gum'/exp OR 'sugarless chewing gum' OR 'sugar near/2 free chewing gum' OR 'chewing gum*':ti OR 'gum chewing':ti OR 'chewing gum*':kw OR 'gum chewing':kw) AND 'diseases'/exp OR 'surgery' OR 'surgical' OR 'post near/2 operative' OR 'peri near/2 operative' OR 'pre near/2 operative' OR 'prevent*' OR 'therap*' OR 'normal human'/exp OR 'quality of life'/exp OR 'wellbeing'/exp OR 'mental performance'/exp OR 'anxiety'/exp OR 'functional stat*' OR 'pregnan*' OR 'delivery' OR 'health outcom*' OR 'anxiety' OR 'patient*'
Scopus	(KEY ((chewing AND gum*) OR (gum AND chewing)) OR TITLE ((chewing AND gum*) OR (gum AND chewing)) OR TITLE-ABS-KEY ((sugarless AND chewing AND gum*) OR (sugar-free AND chewing AND gum*) OR (sugar AND free AND chewing AND gum*))) AND TITLE-ABS-KEY(disease) OR TITLE-ABS-KEY(surgery) OR TITLE-ABS-KEY(surgical) OR TITLE-ABS-KEY(postoperative) OR TITLE-ABS-KEY(perioperative) OR TITLE-ABS-KEY(preoperative) OR TITLE-ABS-KEY(prevent*) OR TITLE-ABS-KEY(therap*) OR TITLE-ABS-KEY(quality of life) OR TITLE-ABS-KEY(wellbeing) OR TITLE-ABS-KEY(mental performance) OR TITLE-ABS-KEY(anxiety) OR TITLE-ABS-KEY(functional stat*) OR TITLE-ABS-KEY(pregnan*) OR TITLE-ABS-KEY(delivery) OR TITLE-ABS-KEY(health outcome) OR TITLE-ABS-KEY (patient*)
PsycINFO	(chewing gum* or gum chewing or sugarless chewing gum or sugar free chewing gum or sugar-free chewing gum).mp. [mp=title, abstract, heading word, table of contents, key concepts, original title, tests & measures, mesh word] AND exp Anxiety/ or anxi*.mp OR exp Cognitive Processes/ or exp Cognitive Ability/ or exp Emotional States/ or mental performance.mp. or exp Mental Health/ or exp Memory/
Cinahl	TI (chewing gum* or gum chewing) OR SU (chewing gum or gum chewing) OR sugarless chewing gum or sugar free chewing gum or sugar-free chewing gum AND disease* or quality of life or wellbeing or pregnancy or surgery or surgical or postoperative or perioperative or preoperative or post-operative or peri-operative or pre-operative or delivery or mental performance or anxiety or prevent* or therap*
