# Supplementary material for: Chewing Gum and Health: A Mapping Review and an Interactive Evidence Gap Map
Source: Nutrients. 2025 Aug 25;17(17):2749. doi: 10.3390/nu17172749 (PMC12430410; doi:10.3390/nu17172749)
Supplement: Supplementary file 1 [file nutrients-17-02749-s001.zip › Table S3.pdf]

Table S3: Included records.

First Author (year)	Title
Yong-Han (2021)	A prospective randomized controlled trial to evaluate effect of chewing gum on postoperative ileus in elderly patient after hip fracture.
Abdelgalil (2023)	Preoperative gum chewing for different durations to prevent postoperative sore throat after endotracheal intubation: A randomized controlled trial
Evans M. Et al.(2018)	Acute Ingestion of Caffeinated Chewing Gum Improves Repeated Sprint Performance of Team Sport Athletes With Low Habitual Caffeine Consumption.
Wickham K.A et al (2018)	Administration of Caffeine in Alternate Forms.
Ahmed (2018)	Efficacy of three different regimens in recovery of bowel function following elective cesarean section: A randomized trial
Ahuja (2024)	Preoperative chewing gum versus pericardial p6 point acupressure for attenuation of postoperative nausea and vomiting in patients undergoing laparoscopic cholecystectomy – A comparative evaluation
Akalpler (2018)	Gum chewing and bowel function after caesarean section under spinal anesthesia
Ali (2019)	Chewing aid in routine postoperative orders-does it reduce postoperative ileus after cesarean section? A randomized control trial
Allen (2015)	Chewing gum: Cognitive performance, mood, well-being, and associated physiology
Allida (2015)	Thirst in chronic heart failure: A review
Allida (2021)	A RandomisEd ControLled TrIal of ChEwing Gum to RelieVE Thirst in Chronic Heart Failure (RELIEVE-CHF)
Altraigey (2020)	The effect of gum chewing on the return of bowel motility after planned cesarean delivery: a randomized controlled trial
Amanda (2019)	Difference of bowel sound return time among post-laparatomy surgery patients after chewing gum
Andersson (2015)	Effects of chewing gum against postoperative ileus after pancreaticoduodenectomy--a randomized controlled trial
Annafi (2021)	Chewing gum administration towards gastrointestinal motility on postoperative patients of PKU Muhammadiyah Gamping Hospital
Anu (2022)	Effect of Mint Flavoured Chewing Gum in Observing Changes in Cognitive Function while Assessing Test Performance-An Interventional Study
Arslan (2024)	Postoperative Ileus and Nonpharmacological Nursing Interventions for Colorectal Surgery: A Systematic Review
Aslan (2025)	Effect of frozen saline and menthol gum on thirst level after abdominal surgery: A mixed-methods study
Best Gregg W.J et al. (2019)	Assessing the effect of sugar-free chewing gum use on the residual gastric volume of patients fasting for gastroscopy: A randomised controlled trial.
Atkins (2022)	Chewing Gum After Radical Cystectomy With Urinary Diversion for Recovery of Intestinal Function: A Systematic Review and Meta-Analysis
Atkinson (2016)	Randomized clinical trial of postoperative chewing gum versus standard care after colorectal resection
Azarpazhooh (2016)	Xylitol for preventing acute otitis media in children up to 12 years of age
Azhar (2016)	Enhanced Recovery after Urological Surgery: A Contemporary Systematic Review of Outcomes, Key Elements, and Research Needs
Aziato (2017)	Labour pain experiences and perceptions: A qualitative study among post-partum women in Ghana
Balabolu (2023)	Effect of Preoperative Carbohydrate Drink and Postoperative Chewing Gum on Postoperative Nausea and Vomiting in Patients Undergoing Day Care Laparoscopic Cholecystectomy: A Randomized Controlled Trial
Bang (2022)	Anxiolytic effects of chewing gum during preoperative fasting and patient-centered outcome in female patients undergoing elective gynecologic surgery: randomized controlled study
Bang (2023)	Effect of chewing gum on anxiety in women undergoing elective cesarean section: a randomized controlled study
Bang (2023)	The effect of adding chewing gum to oral carbohydrates on preoperative anxiety scores in women undergoing gynecological surgery: A randomized controlled study
Beaman (2015)	Want to block earworms from conscious awareness? B(u)y gum!
Bhatti (2021)	Role of Chewing Gum in Reducing Postoperative Ileus after Reversal of Ileostomy: A Randomized Controlled Trial
Boarin (2017)	The use of chewing gum for postoperative ileus prevention in patients undergoing radical cystectomy
Bobillo (2018)	Short-term effects of a green coffee extract-, Garcinia c ambogia- and l-carnitine-containing chewing gum on snack intake and appetite regulation
Bouvet (2017)	Effect of gum chewing on gastric volume and emptying: A prospective randomized crossover study
Bragg (2015)	Postoperative ileus: Recent developments in pathophysiology and management
Brown (2015)	Effect of GutsyGum(tm), A Novel Gum, on Subjective Ratings of Gastro Esophageal Reflux Following A Refluxogenic Meal
Buijs (2018)	Can coffee or chewing gum decrease transit times in Colon capsule endoscopy? A randomized controlled trial
Byrne (2018)	Gum chewing aids bowel function return and analgesic requirements after bowel surgery: a randomized controlled trial
Can (2022)	Effects of gum chewing and repetitive motor activity on sustained attention in adults with attention deficit-hyperactivity disorder
Çevik (2016)	Effect of bed exercises and gum chewing on abdominal sounds, flatulence and early discharge in the early period after caesarean section
Çevik (2025)	The effect of chewing gum on intestinal functions, postoperative pain, and early discharge after isolated coronary bypass surgery
Chae (2024)	Effect of Preoperative Gum Chewing on Postoperative Nausea and Vomiting in Women Undergoing Robotic Laparoscopic Surgery for Uterine Myomas: A Randomized Controlled Trial
Chae (2024)	Impact of Preoperative Gum Chewing on Postoperative Anti-Emetic Use in Robot-Assisted Laparoscopic Surgery for Benign Ovarian Masses: A Prospective, Single-Blinded Randomized Controlled Trial
Chan (2017)	Usage of Chewing Gum in Posterior Spinal Fusion Surgery for Adolescent Idiopathic Scoliosis: A Randomized Controlled Trial
Chan (2020)	Nicotine Replacement Therapy and Healthy Lifestyle Psychoeducation for Smoking Reduction in Acute Psychiatric Inpatients: A Cluster-Randomized Parallel Study
Chapman (2018)	Postoperative ileus following major colorectal surgery
Chen (2023)	Acute enhancement of Romanian deadlift performance after consumption of caffeinated chewing gum.
Chen (2023)	Chewing Gum May Alleviate Degree of Thirst in Patients on Hemodialysis
Chen (2023)	Efficacy and safety of preoperative chewing gum for undergoing elective surgery: A meta-analysis of randomised controlled trials
Weijenberg R. Et al. (2015)	Chew the Pain Away: Oral Habits to Cope with Pain and Stress and to Stimulate Cognition.
Binbin M et al. (2017)	Chewing Gum for Intestinal Function Recovery after Colorectal Cancer Surgery: A Systematic Review and Meta-Analysis.
Ciardulli et al. (2018)	Chewing gum improves postoperative recovery of gastrointestinal function after cesarean delivery: a systematic review and meta-analysis of randomized trials.
Clark (2018)	Early return of bowel function after gynecologic surgery using chewing gum
Schlam et al. (2016)	Comparative effectiveness of intervention components for producing long-term abstinence from smoking: a factorial screening experiment.
Ebrahimian et al. (2022)	Comparison of the effectiveness of virtual reality and chewing mint gum on labor pain and anxiety: a randomized controlled trial.
Ebrahimian A. Et al. (2021)	Comparisons of the Effects of Watching Virtual Reality Videos and Chewing Gum on the Length of Delivery Stages and Maternal Childbirth Satisfaction: A Randomized Controlled Trial.
Darvall (2024)	Chewing gum to treat postoperative nausea and vomiting in female patients: a multicenter randomized trial
de Leede (2018)	Multicentre randomized clinical trial of the effect of chewing gum after abdominal surgery
de Silva (2015)	Effect of gum chewing on air swallowing, saliva swallowing and belching
Dehghanmehr (2018)	Investigating the impact of sugar free gum on the thirst and dry mouth of patients undergoing hemodialysis
Dittrich (2021)	Effects of Caffeine Chewing Gum on Exercise Tolerance and Neuromuscular Responses in Well-Trained Runners.
Douligeris (2023)	The Effect of Postoperative Gum Chewing on Gastrointestinal Function Following Laparoscopic Gynecological Surgery. A Meta-analysis of Randomized Controlled Trials
Doyle (2019)	Chewing gum use in the perioperative period
Du (2021)	Chewing gum promotes bowel function recovery in elderly patients after lumbar spinal surgery: a retrospective single-center cohort study
Dudi-Venkata (2020)	Systematic scoping review of enhanced recovery protocol recommendations targeting return of gastrointestinal function after colorectal surgery
Duluklu (2020)	Effect of Gum Chewing on Recovery after Surgery for Colorectal Surgery Patients: A Randomized Controlled Trial
Ebbert (2015)	Interventions for smokeless tobacco use cessation
Arrabal G. Et al.(2016)	Effect of chewing gum on thirst: an integrative review.
ÇETİNKAYA et al. (2024)	Effect of Chewing Gum and Stress Ball on Labor Pain, Duration of Labor, and Birth Satisfaction: A Randomized Controlled Study.
Esfehani R.J et al (2018)	Effect of Chewing Gum on Post Cesarean Ileus in the North East of Iran: A Randomized Clinical Trial.
Eastwick E. Et al. (2017)	Effect of Gum Chewing on Bowel Motility Following Elective Colon Resection.
Yildizeli T. Et al. (2016)	Effect of gum chewing on reducing postoperative ileus and recovery after colorectal surgery: A randomised controlled trial.
Esmaeelian et al.(2024)	Effect of neroli-flavored chewing gum on anxiety.
Yang C.C et al. (2024)	Effects of Caffeinated Chewing Gum on Exercise Performance and Physiological Responses: A Systematic Review.
van Orten-Luiten et al. (2021)	Effects of Cannabidiol Chewing Gum on Perceived Pain and Well-Being of Irritable Bowel Syndrome Patients: A Placebo-Controlled Crossover Exploratory Intervention Study with Symptom-Driven Dosing.
Jiménez-Ten (2025)	Effects of Chewing Gum on Satiety, Appetite Regulation, Energy Intake, and Weight Loss: A Systematic Review

Yerlikaya-Schatten et al. (2020)	Effects of gum chewing on glycaemic control in women with gestational diabetes mellitus: A randomized controlled trial. Impact of chewing on hyperglycaemia in women with GDM.
Elkan (2022)	The Effect of Xylitol Gum Chewing After Cesarean on Bowel Functions: A Randomized Controlled Study
Emile (2024)	Strategies to reduce ileus after colorectal surgery: A qualitative umbrella review of the collective evidence
Engle (2019)	Effects of motivation phase intervention components on quit attempts in smokers unwilling to quit: A factorial experiment
Fang (2017)	Impact of gum chewing on the quality of bowel preparation for colonoscopy: an endoscopist-blinded, randomized controlled trial
Farmani (2024)	The effect of repeated coffee mouth rinsing and caffeinated gum consumption on aerobic capacity and explosive power of table tennis players: a randomized, double-blind, placebo-controlled, crossover study.
Field (2024)	Caffeine Gum Improves Reaction Time but Reduces Composure Versus Placebo During the Extra-Time Period of Simulated Soccer Match-Play in Male Semiprofessional Players.
Filip-Stachnik (2021)	Effects of acute ingestion of caffeinated chewing gum on performance in elite judo athletes
Flores-Funes (2016)	The use of coffee, chewing-gum and gastrograffin in the management of postoperative ileus: A review of current evidence
Foessleitner (2024)	The Role of hCG and Histamine in Emesis Gravidarum and Use of a Chewing Gum Containing Vitamin C as a Treatment Option: A Double-Blinded, Randomized, Controlled Trial
Fung (2024)	Does Chewing Gum Lead to Earlier Postoperative Gastrointestinal Recovery in Children? A Systematic Review and Meta-analysis
Gabrawi (2022)	Post-tonsillectomy advice: the UK patient experience
Gao (2025)	Effect of chewing gum combined with WeChat-enhanced instruction on bowel preparation in constipated patients: a randomized–controlled trial
Gao (2025)	The effects of coffee vs. Gum chewing after cesarean on bowel functions: a systematic review and network meta-analysis
Garcia (2019)	Menthol chewing gum on preoperative thirst management: Randomized clinical trial
Ge (2015)	Effect of chewing gum on the postoperative recovery of gastrointestinal function
Ge (2017)	Influence of gum-chewing on postoperative bowel activity after laparoscopic surgery for gastric cancer: A randomized controlled trial
Ghorat (2024)	The clinical efficacy of Olibanum gum chewing in patients with Mild-to-Moderate Alzheimer disease: A randomized Parallel-Design controlled trial
Ginns (2019)	Chewing gum while studying: Effects on alertness and test performance
Gong (2015)	Xylitol Gum Chewing to Achieve Early Postoperative Restoration of Bowel Motility after Laparoscopic Surgery
Goudra (2015)	Effect of Gum Chewing on the Volume and pH of Gastric Contents: A Prospective Randomized Study
Göymen (2017)	Effect of gum chewing and coffee consumption on intestinal motility in caesarean sections
Green (2018)	Chewing Gum for Reducing Post-Cesarean Section Ileus.
Guest (2021)	International society of sports nutrition position stand: caffeine and exercise performance
Chan L et al. (2019)	Gum Chewing and Prolonged Postoperative Ileus: An Observational Retrospective Study Examining the Impact of an Evidence-Based Practice Change.
Gungorduk (2021)	Non-pharmacological interventions for the prevention of postoperative ileus after gynecologic cancer surgery
Hables (2025)	Effect of gum chewing and cold therapy on postoperative cesarean women’s self-assessed pain levels and narcotics use: a comparative study
Hamada (2016)	Effect of postprandial gum chewing on diet-induced thermogenesis
Hansson (2019)	Effect of nicotine 6 mg gum on urges to smoke, a randomized clinical trial
Harrison (2015)	Sweet tasting solutions for reduction of needle-related procedural pain in children aged one to 16 years
Hartmann-Boyce (2018)	Nicotine replacement therapy versus control for smoking cessation
Hasegawa (2017)	Flavour-enhanced cortisol release during gum chewing
Hassan (2022)	Effect of Post Cesarean Section Protocol of Care on Early and Follow up Outcomes
Haworth (2024)	Immediate and residual effects of functional chewing gum on sustained attention and mood
Hochner (2015)	Gum chewing and gastrointestinal function following caesarean delivery: A systematic review and meta-analysis
Hsu (2022)	Effects of Gum Chewing on Recovery From Postoperative Ileus: A Randomized Clinical Trail
Hu (2024)	Impact of varied feeding protocols on gastrointestinal function recovery in the early postoperative period following repeat cesarean section: a randomized controlled trial
Huang (2015)	Usefulness of chewing gum for recovering intestinal function after cesarean delivery: A systematic review and meta-analysis of randomized controlled trials
Huang (2020)	The effect of gum consumption on anthropometric characteristics and cardiac disorders: A systematic review and meta-analysis of randomized controlled trials
Huang (2021)	Effectiveness of Improved Use of Chewing Gum During Capsule Endoscopy in Decreasing Gastric Transit Time: A Prospective Randomized Controlled Study
Hung (2021)	Nicotine supplementation enhances simulated game performance of archery athletes.
George K.T (2021)	Improving Outcomes through Implementation of Gum Chewing for Abdominal Surgical Patients.
Mahoney C.R et al. (2019)	Intake of caffeine from all sources and reasons for use by college students.
Iskander (2024)	An outline of the management and prevention of postoperative ileus A review
Jayalal (2025)	EFFICACY OF CHEWING GUM IN REDUCING THE INCIDENCE OF POSTOPERATIVE ILEUS IN PATIENTS UNDERGOING OPEN ABDOMINAL SURGERIES
Jennings (2015)	The use of chewing gum postoperatively in pediatric scoliosis patients facilitates an earlier return to normal bowel function
Jiang (2017)	Sham feeding with chewing gum in early stage of acute pancreatitis: A randomized clinical trial
Kadirogullari (2022)	The effect of chewing gum on bowel function postoperatively in patients with total laparoscopic hysterectomy: a randomised controlled trial
Kamran (2020)	Can adjuncts to bowel preparation for colonoscopy improve patient experience and result in superior bowel cleanliness? A systematic review and meta-analysis
Kanno (2019)	Gum chewing while walking increases fat oxidation and energy expenditure.
Kanza (2021)	Effects of acupressure, gum chewing and coffee consumption on the gastrointestinal system after caesarean section under spinal anaesthesia
Karmali (2015)	Randomized controlled trial of vagal modulation by sham feeding in elective non-gastrointestinal (orthopaedic) surgery
Kaszuba (2023)	The Effect of Caffeinated Chewing Gum on Volleyball-Specific Skills and Physical Performance in Volleyball Players
Kaufeld (2022)	Chewing gum reduces visually induced motion sickness
Kawamura (2024)	Relationship between a gum-chewing routine and oral, physical, and cognitive functions of community-dwelling older adults: A Kashiwa cohort study
Keefe (2018)	Treating pediatric post-tonsillectomy pain and nausea with complementary and alternative medicine
Kim (2019)	A 3-month mastication intervention improves recognition memory
Knapik (2022)	Prevalence of caffeine consumers, daily caffeine consumption, and factors associated with caffeine use among active duty United States military personnel
Kobayashi (2015)	Efficacy of gum chewing on bowel movement after open colectomy for left-sided colorectal cancer: A randomized clinical trial
Koksoy (2025)	The effect of chewing gum on postoperative pain in children undergoing tonsillectomy
Konno (2016)	Relationships Between Gum-Chewing and Stress.
Kresge (2015)	Chewing gum increases energy expenditure before and after controlled breakfasts.
Kruger (2024)	Examining the effects of caffeine during an auditory attention task
Kumar (2023)	A Study of Effect of Motility Chewing Gum in Gut after Abdominal Surgery at NMCH Sasaram
Kusika (2021)	The role of chewing gum on post-operative bowel recovery after gynecological laparoscopic surgery: A short report and updated review
Lee (2016)	Effect of sham feeding with gum chewing on postoperative ileus after liver transplantation—a randomized controlled trial
Lee (2016)	Effects of gum chewing on abdominal discomfort, nausea, vomiting and intake adherence to polyethylene glycol solution of patients in colonoscopy preparation
Lee (2016)	The Role of Xylitol Gum Chewing in Restoring Postoperative Bowel Activity After Cesarean Section
Leung (2019)	Combined nicotine patch with gum versus nicotine patch alone in smoking cessation in Hong Kong primary care clinics: A randomised controlled trial
Lew (2025)	Effect of chewing gum on clinical outcomes and postoperative recovery in adult patients after gastrointestinal surgery: an umbrella review
Liao (2022)	Effects of chewing gum on gastrointestinal function in patients following spinal surgery: a meta-analysis and systematic review
Liao (2024)	Effectiveness of Chewing Gum on Nausea and Vomiting Following Postprocedure: A Systematic Review and Meta-Analysis
Lin (2024)	Efficacy of Electronic Cigarettes vs Varenicline and Nicotine Chewing Gum as an Aid to Stop Smoking A Randomized Clinical Trial
Lindson (2019)	Different doses, durations and modes of delivery of nicotine replacement therapy for smoking cessation
Liu (2017)	Effect of gum chewing on ameliorating ileus following colorectal surgery: A meta-analysis of 18 randomized controlled trials
Liu (2024)	Caffeinated Chewing Gum Improves Basketball Shooting Accuracy and Physical Performance Indicators of Trained Basketball Players: A Double-Blind Crossover Trial
Liu (2025)	Effects of caffeinated chewing gum-induced sympathetic activation and diuretic effect on the rapid rate of weight loss in bodybuilders: a double-blind crossover study
López-Jaimez (2016)	Use of chewing gum in children undergoing an appendectomy: A randomized clinical controlled trial

Lynn (2024)	Caffeine gum improves 5 km running performance in recreational runners completing parkrun events.
Mackeen (2025)	Evidence-based cesarean delivery: postoperative care (part 10)
Mahmoud (2018)	Chewing gum for declining ileus and accelerating gastrointestinal recovery after appendectomy
Malek (2024)	Comparison of the effect of chewing gum with routine method on ileus after burns: a randomized clinical trial
McLellan (2019)	Caffeine and energy drink use by combat arms soldiers in Afghanistan as a countermeasure for sleep loss and high operational demands
Melanson (2017)	Chewing gum decreases energy intake at lunch following a controlled breakfast
Meng (2018)	A Prospective Single-blind Randomized Controlled Trial of Chewing Gum on Bowel Function Recovery After Posterior Spinal Fusion Surgery for Adolescent Idiopathic Scoliosis
Mohsenzadeh-Ledari (2025)	The Effect of Chewing Gum in Managing Labor Pain Intensity and Anxiety Level in Primiparous Women: A Randomized Controlled Trial
Moradi (2022)	The effectiveness of caffeinated chewing gum in ameliorating cognitive functions affected by sleep deprivation
Muhumuza (2025)	The effect of chewing gum on postoperative ileus after laparotomy for gastroduodenal perforations: a randomized controlled trial
Murata (2025)	The Effects of Countermeasures for Preventing Vigilance Decrement During Manual and Automated Driving
Muwel (2024)	Effect of chewing gum in reducing postoperative ileus after gastroduodenal perforation peritonitis surgery: A prospective randomised controlled trial
N (2024)	Chewing Gum Versus Standard Care for Enhanced Bowel Recovery After Cesarean Section: A Randomized Clinical Trial.
Najdi (2018)	Comparison of the effect of misoprostol and chewing gum on intestinal movements after cesarean delivery
Nanthiphatthanachai (2020)	Effect of Chewing Gum on Gastrointestinal Function Recovery After Surgery of Gynecological Cancer Patients at Rajavithi Hospital: A Randomized Controlled Trial
Nascimento (2024)	No Combined Effect of Caffeinated Chewing Gum and Priming Exercise on Oxygen Uptake and Muscle Near-Infrared Spectroscopy-Derived Kinetics: A Double-Blind Randomized Crossover Placebo-Controlled Trial in Cyclists.
Nascimento (2024)	The effect of caffeine chewing gum on muscle performance and fatigue after severe-intensity exercise: isometric vs. dynamic assessments in trained cyclists
Naz (2023)	Effects of menthol gum chewing on postoperative nausea, vomiting, and length of hospital stay in children undergoing appendectomy: A randomized controlled trıal
Nezakati (2018)	The comparison of effect of abdominal massage and chewing sugar-free gum on the incidence and severity of constipation in male patients undergoing skeletal traction of lower limbs: A single-blind clinical trial
Nishigawa (2015)	Masticatory performance alters stress relief effect of gum chewing
Oberlin-Brown (2016)	Oral Presence of Carbohydrate and Caffeine in Chewing Gum: Independent and Combined Effects on Endurance Cycling Performance.
Öbrink (2019)	Can simple perioperative measures improve quality of recovery following ambulatory laparoscopic surgery in females? An open prospective randomised cohort study, comparing nutritional preoperative drink and chewing gum during recovery to standard care
Okgün (2025)	Does Preoperative Gum Chewing Reduce Postoperative Sore Throat?
Ong (2022)	Effects of Chewing Gum on Nitric Oxide Metabolism, Markers of Cardiovascular Health and Neurocognitive Performance after a Nitrate-Rich Meal
Ouanes (2015)	The role of perioperative chewing gum on gastric fluid volume and gastric pH: A meta-analysis
Özkan (2025)	Efficacy of Chewing Xylitol Gum on Restoring Postoperative Bowel Activity After Laparoscopic Cholecystectomy: A Three-arm Randomized Controlled Trial
Pan (2017)	Gum chewing combined with oral intake of a semi-liquid diet in the postoperative care of patients after gynaecologic laparoscopic surgery
Pang (2018)	Prospective Implementation of Enhanced Recovery After Surgery Protocols to Radical Cystectomy
Park (2016)	Short-term effects of chewing gum on satiety and afternoon snack intake in healthy weight and obese women
Park (2018)	Meta-Analysis of the Effect of Gum Chewing After Gynecologic Surgery
Pattamatta (2018)	Health-related quality of life and cost-effectiveness analysis of gum chewing in patients undergoing colorectal surgery: results of a randomized controlled trial
Pereira (2016)	Chewing gum for enhancing early recovery of bowel function after caesarean section
Pham (2020)	Nicotine enhances auditory processing in healthy and normal-hearing young adult nonsmokers.
Murad et al.(2022)	Pharmaceutical interventions: A solution to stop smoking.
Pilevarzadeh (2016)	Effect of gum chewing in the reduction of paralytic ileus following cholecystectomy
Pirmohammadi (2023)	Early absorption sources of caffeine can be a useful strategy for improving female table tennis players-specific performance.
Little P. Et al. (2017)	Probiotic capsules and xylitol chewing gum to manage symptoms of pharyngitis: a randomized controlled factorial trial.
Quirk (2018)	Supportive interventions to improve physiological and psychological health outcomes among patients undergoing cystectomy: A systematic review
Ranchordas (2018)	Effects of Caffeinated Gum on a Battery of Soccer-Specific Tests in Trained University-Standard Male Soccer Players.
Ranchordas (2019)	Effect of caffeinated gum on a battery of rugby-specific tests in trained university-standard male rugby union players
Roldan (2022)	Enhanced Recovery After Surgery reduced length of stay after colorectal surgery in a small rural hospital in Ontario
Roslan (2020)	The Impact of Sham Feeding with Chewing Gum on Postoperative Ileus Following Colorectal Surgery: a Meta-Analysis of Randomised Controlled Trials
Russell (2020)	Physiological and Performance Effects of Caffeine Gum Consumed During a Simulated Half-Time by Professional Academy Rugby Union Players.
Sammut (2021)	The effect of gum chewing on postoperative ileus in open colorectal surgery patients: A review
Sarmiento-Altamirano (2025)	Reduction of postoperative ileus in gastrointestinal surgery: systematic review and meta-analysis
Sayilan (2020)	The Effect of Gum Chewing on Abdominal Pain and Nausea Caused by Polyethylene Glycol Solution Used for Intestinal Cleansing before Colonoscopy: An Endoscopist-Blinded, Randomized Controlled Trial
Schlam (2018)	Can we increase smokers' adherence to nicotine replacement therapy and does this help them quit?
Shekari (2022)	Bright light alone or combined with caffeine improves sleepiness in chronically sleep-restricted young drivers
Shiffman (2020)	Effectiveness of nicotine gum in preventing lapses in the face of temptation to smoke among non-daily smokers: a secondary analysis
Shiffman (2020)	Using nicotine gum to assist nondaily smokers in quitting: A randomized clinical trial
Shiu (2024)	Acute ingestion of caffeinated chewing gum reduces fatigue index and improves 400-meter performance in trained sprinters: a double-blind crossover trial
Shiu (2024)	Caffeinated chewing gum improves the batting and pitching performance of female softball players: a randomized crossover study.
Short (2015)	Chewing gum for postoperative recovery of gastrointestinal function
Shum (2016)	Randomized clinical trial of chewing gum after laparoscopic colorectal resection
Singh (2023)	Effect of Chewing Gum on Recovery of Intestinal Function after Caesarean Section
Sinz (2023)	Gum Chewing and Coffee Consumption but not Caffeine Intake Improve Bowel Function after Gastrointestinal Surgery: a Systematic Review and Network Meta-analysis
Loreto A.R et al. (2016)	Smoking Cessation Treatment for Patients With Mental Disorders Using CBT and Combined Pharmacotherapy.
Song (2016)	Meta-analysis comparing chewing gum versus standard postoperative care after colorectal resection
Arrabal G et al. (2019)	Strategies for thirst relief: integrative literature review.
Su'a (2015)	Chewing gum and postoperative ileus in adults: A systematic literature review and meta-analysis
Tahir (2018)	Multicentre observational study of gastrointestinal recovery after elective colorectal surgery
Tallis (2024)	Are caffeine effects equivalent between different modes of administration: the acute effects of 3 mg.kg-1 caffeine on the muscular strength and power of male university Rugby Union players
Tazegül (2015)	Gum chewing reduces the time to first defaecation after pelvic surgery: A randomised controlled study
Teimouri-Korani (2025)	Individual responses to encapsulated caffeine and caffeine chewing gum on strength and power in strength-trained males
Tharwat (2021)	Impact of sugarless chewing gum versus peppermint on first bowel movement after cesarean section: Randomized controlled trial
Al-kharabsheh M et al. (2023)	The Effect of Chewing Sugar-Free Gum to Improve Bowel Movement After Colorectal Surgeries in Patients With Colorectal Cancer.
Kartal T Et al. (2024)	The Effect of Chewing Gum on Stress in Women with Unexplained Infertility.
Sahin E. Et al. (2015)	The Effect of Gum Chewing, Early Oral Hydration, and Early Mobilization on Intestinal Motility After Cesarean Birth.
Asakawa et al. (2020)	The Effects of Chewing Gum in Preventing Eyestrain.
Whalley P.J et al. (2015)	The Effects of Different Forms of Caffeine Supplement on 5-km Running Performance.
Siti Z.MZ. et al. (2021)	The Effects of Preoperative Sugarless Gum Chewing on Gastric Fluid Volume and pH.
Jian T.L et al. (2016)	The Role of Xylitol Gum Chewing in Restoring Postoperative Bowel Activity After Cesarean Section.
Tong (2023)	Chewing Gum Cannot Reduce Postoperative Abdominal Pain and Nausea After Posterior Spinal Fusions in Patients With Adolescent Idiopathic Scoliosis: A Systematic Review and Meta-analysis of Randomized Controlled Trials
Tsai (2024)	Effects of Caffeinated Chewing Gum on Ice Hockey Performance after Jet Lag Intervention: Double-Blind Crossover Trial
Turkay (2020)	The impact of chewing gum on postoperative bowel activity and postoperative pain after total laparoscopic hysterectomy
Tuscharoenporn (2024)	Effects of Postoperative Gum Chewing on Recovery of Gastrointestinal Function Following Laparoscopic Gynecologic Surgery: Systematic Review and Meta-Analysis of Randomized Controlled Trials

Ulasi (2023)	The Effects of Combined Gum-chewing and Parenteral Metoclopramide on the Duration of Postoperative Ileus After Abdominal Surgery.
Umeda (2016)	Effects of caffeinated chewing gum on muscle pain during submaximal isometric exercise in individuals with fibromyalgia
Urcanoglu (2021)	Effects of Gum Chewing on Early Postoperative Recovery After Laparoscopic Cholecystectomy Surgery: a Randomized Controlled Trial
Valencia (2019)	Chewing gum for 1 h does not change gastric volume in healthy fasting subjects. A prospective observational study
Van Den Heijkant (2015)	Randomized clinical trial of the effect of gum chewing on postoperative ileus and inflammation in colorectal surgery
Venier (2019)	Acute Enhancement of Jump Performance, Muscle Strength, and Power in Resistance-Trained Men After Consumption of Caffeinated Chewing Gum
Vergara-Fernandez (2016)	Usefulness of Gum Chewing to Decrease Postoperative Ileus in Colorectal Surgery with Primary Anastomosis: A Randomized Controlled Trial
Vuletic (2017)	The effect of chewing a sugar-free gum after oatmeal on the postprandial glycaemia - A cross-over study
Wagner (2022)	The Problem of Appetite Loss after Major Abdominal Surgery: A Systematic Review
Walker (2016)	Chewing unflavored gum does not reduce cortisol levels during a cognitive task but increases the response of the sympathetic nervous system
Wang (2020)	Effects of Preoperative Gum Chewing on Sore Throat After General Anesthesia With a Supraglottic Airway Device: A Randomized Controlled Trial
Wardhani (2020)	Effects of nicotine on pupil size and performance during multiple-object tracking in non-nicotine users
Wefer (2024)	Non-pharmacological interventions to reduce thirst in patients with heart failure or hemodialysis: A systematic review and meta-analysis
Wen (2017)	Chewing gum for intestinal function recovery after caesarean section: A systematic review and meta-analysis
Won (2021)	Effects of chewing gum stimuli on general and emotional stroop test
Xu (2015)	The effect of gum chewing on blood GLP-1 concentration in fasted, healthy, non-obese men
Xu (2018)	Effect of chewing gum on gastrointestinal function after gynecological surgery: A systematic literature review and meta-analysis
Yalazı (2025)	The Effects of Chewing Gum and Drinking Fennel Tea on Intestinal Motility Post-Cesarean Section: A Randomized Controlled Trial
Yaman-Sözbir (2019)	Effect of chewing gum on stress, anxiety, depression, self-focused attention, and academic success: A randomized controlled study
Yamanaka-Kohno (2024)	Perioperative gum-chewing training prevents a decrease in tongue pressure after esophagectomy in thoracic esophageal cancer patients: a nonrandomized trial
Yang (2017)	Comparison of efficacy of simo decoction and acupuncture or chewing gum alone on postoperative ileus in colorectal cancer resection: a randomized trial
Yang (2018)	Chewing xylitol gum could accelerate bowel motility recovery after elective open proctectomy for rectal cancer
Yenigul (2020)	Efficacy of chewing gum for improving bowel function after cesarean sections: a randomized controlled trial
Yildizeli (2020)	Effect of Gum Chewing on Pain and Anxiety in Turkish Children During Intravenous Cannulation: A Randomized Controlled Study
Yin (2023)	Association of gum chewing with early gastrointestinal recovery in single-port laparoscopic gynecologic surgery
Yin (2023)	The impact of gum-chewing on postoperative ileus following gynecological cancer surgery: A systematic review and meta-analysis of randomized controlled trials
Yıldızeli (2024)	A Contributing Approach to The Management of Pain and Anxiety Associated with Tube Thoracostomy: Chewing Gum
Zainab (2020)	CHEWING GUM TO HASTEN BOWEL RECOVERY IN CESAREAN SECTION: A RANDOMIZED CONTROL TRIAL
Zhang (2025)	Efficacy and safety of therapeutic means for postoperative ileus: an umbrella review of meta-analyses
Zhou (2023)	Efficacy and safety of preoperative chewing gum for undergoing elective surgery: A meta-analysis of randomised controlled trials.
Zou (2024)	Effect of chewing gum of different weights before surgery on sore throat after total thyroidectomy: A randomized controlled trial
